# Interaction Between Vitamin D Metabolism Genetic Variants: Association with Hypovitaminosis D, Rheumatoid Arthritis, and Its Clinical Disease Activity

**DOI:** 10.3390/genes16080967

**Published:** 2025-08-18

**Authors:** Bertha Campos-López, Melissa Rivera-Escoto, Adolfo I. Ruiz-Ballesteros, Karen Pesqueda-Cendejas, Paulina E. Mora-García, Mónica R. Meza-Meza, Isela Parra-Rojas, José M. Moreno-Ortíz, Eneida Turiján-Espinoza, Juan M. Vargas-Morales, Sergio Cerpa-Cruz, Ulises De la Cruz-Mosso

**Affiliations:** 1 Red de Inmunonutrición y Genómica Nutricional en las Enfermedades Autoinmunes; Departamento de Neurociencias, Centro Universitario de Ciencias de la Salud, Universidad de Guadalajara, Guadalajara 44340, Jalisco, Mexico; 2 Instituto de Neurociencias Traslacionales, Departamento de Neurociencias, Centro Universitario de Ciencias de la Salud, Universidad de Guadalajara, Guadalajara 44340, Jalisco, Mexico; 3 Doctorado en Ciencias en Biología Molecular en Medicina, Departamento de Biología Molecular y Genómica, Centro Universitario de Ciencias de la Salud, Universidad de Guadalajara, Guadalajara 44340, Jalisco, Mexico; 4 Laboratorio de Investigación en Obesidad y Diabetes, Facultad de Ciencias Químico-Biológicas, Universidad Autónoma de Guerrero, Chilpancingo de los Bravo 39087, Guerrero, Mexico; 5 Instituto de Genética Humana “Dr. Enrique Corona Rivera”, Departamento de Biología Molecular y Genómica, Centro Universitario de Ciencias de la Salud, Universidad de Guadalajara, Guadalajara 44340, Jalisco, Mexico; 6 Laboratorio de Inmunología y Biología Celular y Molecular, Facultad de Ciencias Químicas, Universidad Autónoma de San Luis Potosi, San Luis Potosi 78210, San Luis Potosi, Mexico; 7 Departamento de Medicina Traslacional y Molecular, Centro de Investigación en Ciencias de la Salud y Biomedicina (CICSaB), Universidad Autónoma de San Luis Potosi, San Luis Potosi 78210, San Luis Potosi, Mexico; 8 Departamento de Reumatología, O.P.D. Hospital Civil de Guadalajara Fray Antonio Alcalde, Guadalajara 44280, Jalisco, Mexico

**Keywords:** vitamin D receptor, vitamin D genes, *CYP27B1*, genetic variants, vitamin D, hypovitaminosis D, multifactor dimensionality reduction (MDR), rheumatoid arthritis

## Abstract

Background: Hypovitaminosis D has been associated with worse rheumatoid arthritis (RA) manifestations. Notably, different genetic studies have reported that approximately 65% of hypovitaminosis D can be partially explained using the presence of single-nucleotide variants (SNVs) in key genes involved in its metabolism. This study aimed to investigate the association and gene–gene interactions of four SNVs in vitamin D metabolism genes, rs10741657 (*CYP2R1*), rs10877012 (*CYP27B1*), rs4809959 *(CYP24A1*), and rs731236 *TaqI* (*VDR*)*,* with hypovitaminosis D, RA, and its clinical disease activity in a Mexican mestizo population. Methods: This study was conducted among females: 204 RA patients and 204 control subjects (CS). Vitamin D serum levels (calcidiol) were analyzed using ELISA, SNVs through allelic discrimination with TaqMan^®^ probes, and were analyzed using a multifactor dimensionality reduction (MDR) method. Results: MDR analysis suggested that GG and TT genotypes of rs10877012 (*CYP27B1*) were linked to lower calcidiol levels, while the CT and CC genotypes of rs731236 *TaqI* (*VDR*) were associated with increased RA susceptibility and higher disease activity. Logistic regression confirmed that the GG genotype of rs10877012 (*CYP27B1*) was associated with hypovitaminosis D (OR = 1.8; CI: 1.1–3.0; *p* = 0.01), and the CT genotype of rs731236 *TaqI* (*VDR*) with RA (OR = 1.9; CI: 1.2–2.9; *p *< 0.01) and high DAS28-ESR (OR = 3.6; CI: 1.3–10.7; *p* < 0.01). Conclusions: The GG genotype of rs10877012 *CYP27B1* was associated with susceptibility to hypovitaminosis D, whereas the CT genotype of rs731236 *TaqI VDR* confers susceptibility to RA and high clinical disease activity in the Mexican mestizo population.

## 1. Introduction

Rheumatoid arthritis (RA) is an autoimmune disease with a multifactorial etiology and heterogeneous clinical manifestations. Its development is strongly linked to genetic background, with a heritability of around 60%. Moreover, the interaction between genetic variants and environmental factors could have an epistatic, cumulative effect on disease evolution and comorbidities in RA patients [1]. Among the environmental susceptibility factors for the disease, hypovitaminosis D is more frequent in RA patients compared to the healthy population. Furthermore, hypovitaminosis D has been associated with higher RA clinical disease activity [2]. Different genetic association studies have reported that around 65% of hypovitaminosis D can be partially explained by the presence of single nucleotide variant (SNV) genetic polymorphisms in key genes of its metabolism [3], such as *CYP2R1*, which encodes the 25-hydroxylase enzyme that catalyzes the conversion of vitamin D precursors into calcidiol, *CYP27B1*, which encodes the 1-α hydroxylase enzyme responsible for conversion of calcidiol into the active metabolite of vitamin D calcitriol, *CYP24A1*, which encodes the 24-hydroxylase that negatively regulates serum levels of calcidiol–calcitriol, and *VDR*, which encodes the vitamin D receptor (VDR). SNVs in these genes have been associated in Caucasian and Asian populations with a higher prevalence of hypovitaminosis D and higher severity of RA manifestations; nevertheless, they have not been previously studied in the Mexican population with RA [4,5]. Particularly, calcitriol exerts immunomodulatory effects through binding to the VDR, a nuclear transcription factor expressed in various immune cell subsets, including dendritic cells, macrophages, and T lymphocytes [6,7]. Upon activation by calcitriol, VDR translocates to the nucleus and binds to vitamin D response elements (VDREs) in the promoter regions of target genes, regulating transcriptional programs involved in immune tolerance and cytokine balance [7,8]. Notably, genes encoding enzymes involved in the metabolism of vitamin D (*CYP2R1*, *CYP27B1*, and *CYP24A1*) harbor SNVs that have been associated with alterations in circulating vitamin D levels, immune cell function, and susceptibility to autoimmune diseases such as rs10741657 [11:14893332 (GRCh38)] *CYP2R1*, rs10877012 [12:57768302 (GRCh38)] *CYP27B1*, rs4809959 [20:54169320 (GRCh38)] *CYP24A1*, and rs731236 C>T [12:47844974 (GRCh38)] *TaqI VDR* [3,9,10]. Specifically, rs10877012 in *CYP27B1*, located in the 5′ untranslated region (UTR), has been shown to modulate gene expression through cis-regulatory mechanisms [10]. The G allele of this variant has been associated with reduced promoter activity, diminished conversion of calcidiol to calcitriol [10], and impaired immunoregulation, which may predispose individuals to chronic inflammation and autoimmunity [11,12,13]. Regarding the genomic and non-genomic effects of vitamin D, the VDR gene, located on chromosome 12q13.11, contains several polymorphic sites with potential functional relevance [14]. One of the most studied is the rs731236 *TaqI VDR* variant, located in exon 9 near the 3′ UTR [12:47844974 (GRCh38)]. Although this is a synonymous SNV that does not alter the amino acid sequence, it is in linkage disequilibrium with other functional variants located in the 3′UTR of *VDR*, such as the VNTR “Singlet A”. These variants may influence the polyadenylation signal, affect mRNA isoform stability, and ultimately modulate receptor expression levels [14,15]. These post-transcriptional modifications can alter VDR bioavailability and responsiveness to calcitriol, thereby influencing immune modulation and autoimmunity susceptibility. Although several studies have explored the individual effects of SNVs in vitamin D-related genes, few have assessed their potential interactive and epistatic effects on RA pathogenesis and hypovitaminosis D in populations with admixed genetic ancestry. In this context, multifactor dimensionality reduction (MDR) is a powerful analytical strategy that allows detection of non-linear interactions among multiple genetic variants without assuming specific genetic models for studying functionally connected but genomically unlinked loci, such as those involved in vitamin D biosynthesis, activation, and signaling [16]. Therefore, this study aimed to investigate the associations and gene–gene interactions of four SNVs in vitamin D metabolism genes, rs10741657 (*CYP2R1*), rs10877012 (*CYP27B1*), rs4809959 (*CYP24A1*), and rs731236 *TaqI* (*VDR*) with hypovitaminosis D, RA, and its clinical disease activity in a Mexican mestizo population.

## 2. Materials and Methods

### 2.1. Subjects

A cross-sectional study was conducted involving 408 females divided into 204 RA patients and 204 control subjects (CS). RA patients were classified according to the ACR/EULAR criteria [17,18] and recruited from 2023–2025 in the Rheumatology Department of the Hospital Civil Fray Antonio Alcalde in Guadalajara, Jalisco, Mexico. RA patients included in this study reported no pregnancy, trauma, recent infections, surgery, or other autoimmune conditions. Regarding CS, they and their relatives had no family history of autoimmunity. All participants were classified as a Mexican mestizo population, with family ancestry of at least three generations born in Western Mexico [19].

### 2.2. Ethical Considerations

This study was approved by the Research Ethical Committee of the Hospital Civil Fray Antonio Alcalde (Approval Code: CEI. 135/23) on 4 May 2023, according to international research ethics guidelines. Before enrollment, all participants signed an informed consent.

### 2.3. Clinical and Biochemical Evaluation

Demographic variables and current treatment of RA patients were recorded. Clinical disease activity was assessed by the 28-joint Disease Activity Score using Erythrocyte Sedimentation Rate [DAS-28 (ESR)] criteria [20]. For biochemical variables, serum was obtained from 12 hour-fasting blood sample. Glucose, lipid profile (triglycerides, total cholesterol, HDL-C, and LDL-C), C-reactive protein, uric acid, and albumin were assessed using a Clinical Chemistry Analyzer (Mindray-BS-240, Shenzhen, China) and spectrophotometric and turbidimetric assays (BioSystems^®^ kits, Barcelona, Spain).

### 2.4. Calcidiol Quantification

Calcidiol serum levels were measured using a spectrophotometer (Multiskan GO, Thermo Scientific^TM^ 51119000, Waltham, MA, USA). Calcidiol (25-hydroxy-vitamin D) quantification was made with the human soluble 25-OH Vitamin D ELISA Kit, Eagle Biosciences^®^, VID31-K01, Amherst, NH, USA. The cutoff values for serum calcidiol levels were as follows: with hypovitaminosis D (<20 ng/mL) and without hypovitaminosis D (≥20 ng/mL) [21].

### 2.5. Genotyping of SNVs in Vitamin D Metabolism Genes

Genomic DNA was extracted from peripheral blood leukocytes using the salting-out method [22]. Genotyping was done with genomic DNA extracted from total leukocytes using the allelic discrimination method. Polymerase chain reaction (PCR) was carried out in MicroAmp^®^Fast Optical 96-Well Reaction Plate with Barcode (0.1 mL) (Applied Biosystems Foster City, CA, USA^®^) on a LightCycler^®^ 96 real-time PCR system (Roche Diagnostics, Indianapolis, IN, USA) as follows: 95 °C for 10 min, followed by 40 cycles of 95 °C for 15 s and 60 °C for 1 min. The rs10741657 (*CYP2R1*) (A>G, rs10741657, C___2958430_10), rs10877012 (*CYP27B1*) (G>T, rs10877012, C__26237740_10), rs4809959 (*CYP24A1*) (A>G, rs4809959, C__27923836_10) and rs731236 *TaqI* (*VDR*) (C>T, rs731236, C___2404008_10) were identified using TaqMan^TM^ pre-designed genotyping assays (Applied Biosystems; Foster City, CA, USA^®^). For each genotype, VIC homozygous, FAM homozygous, and VIC/FAM heterozygous reference controls were included.

### 2.6. Statistical Analysis

Statistical analysis was performed using STATA v.15 (College Station, TX, USA) and GraphPad Prism v 8.0 (San Diego, CA, USA) software, and statistical power was evaluated according to sample size calculations using the Fleiss formula for case–control studies [23]. According to allele frequencies reported in the 1000 genomes study for rs10741657 in *CYP2R1*, rs10877012 in *CYP27B1*, rs4809959 in *CYP24A1*, and rs731236 *TaqI* in *VDR* SNVs in the Latin American population 2 (with predominantly European and Native American ancestry) [24], a sample of 73 subjects was necessary to find the susceptibility alleles and genotypes described previously in the Mexican mestizo population. The Shapiro–Wilk test was applied for the normality test. Descriptive categorical variables were reported as frequencies, and continuous variables with nonparametric variables as medians and percentiles 5th–95th. We determined the genotype and allele frequencies of the SNVs by direct counting and performed the χ^2^ test to compare proportions between groups and to evaluate Hardy–Weinberg equilibrium (HWE). For nonparametric quantitative determinations between two groups, the Mann–Whitney U test was used, and for nonparametric quantitative determinations between three groups, the Kruskal–Wallis test was applied. Odds ratios (OR) with 95% confidence intervals (CI) were used to analyze the potential susceptibility of SNVs in vitamin D metabolism genes with hypovitaminosis D, RA, and clinical activity susceptibility. Gene–gene interactions among the four SNVs were estimated in multifactor dimensionality reduction (MDR) software (version 3.0.2) [16]. The best combination of the SNVs was selected with the ReliefF filter algorithm. Differences were considered statistically significant with a *p*-value < 0.05.

## 3. Results

### 3.1. Anthropometric, Biochemical, Clinical, and Vitamin D Variables from RA Patients and CS

A total of 408 females, divided into 204 RA patients and 204 CS, were assessed. RA patients had a median age of 48 (28–65) years, while CS had a median age of 34 (19–59) (*p *< 0.001). RA patients have a higher BMI (RA = 27.2 kg/m^2^ vs. CS = 24.0 kg/m^2^; *p *< 0.001). Additionally, RA patients have higher serum levels of triglycerides (RA = 98.5 mg/dL vs. CS = 76.0 mg/dL; *p *< 0.001) and lower levels of HDL (RA = 48.9 mg/dL vs. CS = 51.4 mg/dL; *p *= 0.02), LDL (RA = 92.1 mg/dL vs. CS = 95.2 mg/dL; *p *< 0.01), and uric acid (RA = 3.8 mg/dL vs. CS = 4.4 mg/dL; *p *< 0.001) than CS. On the other hand, RA patients had a median disease duration of 7 years and a clinical activity score of 3.5 points by DAS28-ESR; according to the categorized clinical activity (DAS28-ESR > 2.6) of the disease, 79% of RA patients presented activity, and 21% remission (DAS28-ESR < 2.6). Likewise, serum CRP levels were higher in RA (RA = 4.9 mg/dL vs. CS = 1.2 mg/dL; *p *< 0.001) than in CS. Regarding stratified serum vitamin D (calcidiol) levels, 63% of patients presented hypovitaminosis D, and similarly to CS with 64%. Regarding treatment for disease control, 68% of RA patients evaluated received NSAIDs, 18% glucocorticoids, 85% methotrexate, and 71% sulfasalazine (Table 1).

### 3.2. Interaction of Vitamin D Metabolism SNVs with Hypovitaminosis D, RA, and Its Clinical Disease Activity Susceptibility

A genotype–genotype susceptibility interaction analysis was performed that included the genetic variants rs10741657 in *CYP2R1*, rs10877012 in *CYP27B1*, rs4809959 in *CYP24A1*, and rs731236 in *VDR* for the following three scenarios: to assess the susceptibility of developing hypovitaminosis D, developing RA, and greater clinical activity of the disease.

**Figure 1:**
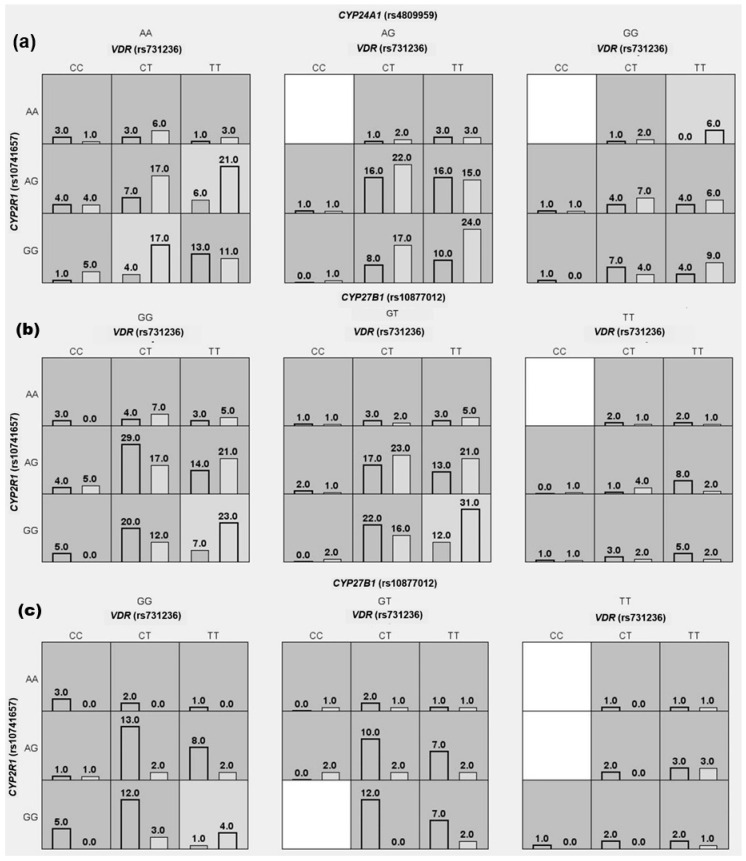
Three-locus interaction of variants in vitamin D metabolism genes for hypovitaminosis D, RA, and its clinical disease activity susceptibility. (**a**) Susceptibility to hypovitaminosis D. Deficiency cases (left bar) and non-deficiency cases (right bar). (**b**) Susceptibility to RA. RA cases (left bar) and CS (right bar). (**c**) Susceptibility to RA clinical activity. Active disease (left bar) and remission (right bar). Multifactor Dimensionality Reduction (MDR) analysis. Each panel represents the interaction between combinations of SNVs in genes involved in vitamin D metabolism: rs10741657 in *CYP2R1*, rs4809959 in *CYP24A1*, rs10877012 in *CYP27B1*, and rs731236 *TaqI* in *VDR*. Left bars in each cell represent the percentage of RA patients; right bars represent CS. Cells shaded in dark gray and light gray denote genotype combinations classified as high-susceptibility and low susceptibility, respectively, based on the ratio of RA patients to CS. Blank cells indicate that genotype combinations are not present in the data.

In the first scenario, hypovitaminosis D (calcidiol < 20 ng/mL) was evaluated in RA patients and CS. Three interaction models were identified; the first consisted of the interaction of SNVs rs731236 in *VDR*, rs10741657 in *CYP2R1*, and rs4809959 in *CYP24A1*. The second model consisted of the interaction of rs10877012 in *CYP27B1* and rs4809959 in *CYP24A1*. Finally, a third model discriminated against the SNV with the highest probability of conferring susceptibility to hypovitaminosis D of all the variants analyzed; this SNV was rs10877012 in *CYP27B1*. Specifically, carrying the GG or TT genotype for the rs10877012 variant in *CYP27B1* confers a 1.7-fold increased susceptibility to hypovitaminosis D (OR = 1.7; CI = 1.1–2.7; TA = 0.57; CVC = 10/10; *p *= 0.02) compared with carriers of the GT genotype or being a carrier of any other SNVs analyzed (Table 2, Figure 1a).

In the second scenario, the susceptibility of RA was evaluated, and three interaction models were identified. The first consisted of the interaction of SNVs rs731236 in *VDR*, rs10741657 in *CYP2R1*, and rs10877012 in *CYP27B1*; the second consisted of the interaction of rs731236 in *VDR* and rs10877012 in *CYP27B1*. Finally, the third model that discriminated against the SNV most likely to suggest a potential genetic susceptibility to RA from the evaluated variants was rs731236 in *VDR*. Particularly, carrying the CT or CC genotype for the rs731236 variant confers 1.9-fold greater susceptibility to the disease (OR = 1.9; CI = 1.3–2.8; TA = 0.58; CVC = 10/10; *p* < 0.01) compared with carriers of the TT genotype or carriers of any other SNVs analyzed (Table 2, Figure 1b).

Finally, in the third scenario, in which disease activity was assessed using the DAS28-ESR index, three interaction models were identified; the first consisted of the interaction of SNVs rs731236 in *VDR*, rs10741657 in *CYP2R1*, and rs10877012 in *CYP27B1*. The second consisted of the interaction between rs731236 in *VDR* and rs10741657 in *CYP2R1*. The third model that discriminated against the SNV most likely to suggest a potential genetic susceptibility of presenting a higher disease activity score from the evaluated variants was rs731236 in *VDR*. Specifically, carrying the CT or CC genotype for the rs731236 variant in *VDR* confers 2.8-fold greater susceptibility to present greater disease activity (OR = 2.8; CI = 1.2–6.6; TA = 0.62; CVC = 10/10; *p* = 0.01) compared with carriers of the TT genotype or any other SNVs analyzed (Table 2, Figure 1c).

### 3.3. Genotypic and Allelic Frequencies of SNV from Vitamin D Metabolism Genes in RA Patients and CS

The distribution of genotypic and allelic frequencies was for rs10741657 *CYP2R1*, the most frequent genotype in both RA patients and CS was AG (47% in both groups), followed by GG (RA: 41% vs. CS: 43%) and AA (RA: 12% vs. CS: 10%) (Table 3). The G allele was the most prevalent (RA: 65% vs. CS: 66%), followed by allele A (RA: 35% vs. CS: 34%). Genotype frequencies in the control group were in HWE (χ^2^ = 0.3; *p* = 0.6)(. For rs10877012 *CYP27B1*, the most frequent genotype in patients with RA was the GG genotype (RA: 47% vs. CS: 44%), while in the CS group, the most frequent genotype was GT (RA: 41% vs. CS: 49%). However, the TT genotype was found at a lower frequency in both study groups (RA: 12% vs. CS: 7%) (Table 3). The most frequent allele in both study groups was G allele (RA: 68% vs. CS: 68%), followed by the T allele (RA: 32% vs. CS: 32%). The population was in the HWE (χ^2^ = 1.8; *p* = 0.17). Regarding rs46400959 *CYP24A1*, the most frequent genotype in patients with RA was the AA genotype (RA: 43% vs. CS: 36%), while in the CS group the most frequent genotype was AG (RA: 34% vs. CS: 42%), however, in both study groups the GG genotype was found at a lower frequency (RA: 23% vs. CS: 22%) (Table 3). The most frequent allele in both study groups was A allele (RA: 60% vs. CS: 57%), followed by G allele (RA: 40% vs.CS: 43%). The population was in HWE (χ^2^ =2.0; *p* = 0.15), and for the rs731236 *TaqI VDR* the most frequent genotype in the RA was the CT genotype (RA: 54% vs. CS: 41%), followed by the TT genotype (RA: 38% vs. CS: 54%), and less frequently the CC genotype (RA: 8% vs. CS: 5%) (Table 3). The most frequent allele in the RA was the T allele (RA: 65% vs. CS: 74%), followed by the C allele (RA: 35% vs. CS: 26%). Genotypic frequencies in CS were consistent with HWE (χ^2^ = 0.6; *p* = 0.42).

### 3.4. Association of SNVs from Vitamin D Metabolism Genes with Hypovitaminosis D, RA, and Its Clinical Disease Activity Susceptibility

Genotype frequencies of each variant were analyzed independently across three different scenarios: hypovitaminosis D, RA susceptibility, and clinical activity using logistic regression models. It was observed that carrying the GG genotype (OR = 1.8; CI = 1.1–3.0; *p* = 0.01) in rs10877012 in *CYP27B1* was associated with higher susceptibility to hypovitaminosis D. On the other hand, for the susceptibility of RA, carrying the CT genotype (OR = 1.9; CI = 1.2–2.9; *p* < 0.01) in rs731236 *VDR* was associated with higher susceptibility to the disease. Additionally, carrying the CT genotype (OR:3.6; CI = 1.3–10.7; *p* < 0.01) in rs731236 *VDR* was associated with higher susceptibility to clinical disease activity. These results are consistent with those obtained from the MDR analysis (Table 3).

## 4. Discussion

The pathogenesis of RA is determined by a complex interplay of genetic, epigenetic, environmental, and immunometabolic factors, including alterations in vitamin D homeostasis; notably, patients with autoimmune diseases often exhibit hypovitaminosis D compared with healthy subjects [25,26]. These multifactorial processes result from the cumulative effects of multiple genetic variants acting within interconnected immunometabolic networks. Among these, SNVs in key genes involved in the vitamin D metabolism pathway have gained relevance, as genome-wide association studies (GWAS) and case-control studies have identified specific loci significantly associated with both susceptibility to RA and modulation of vitamin D levels across different populations [3,9,27]. In particular, four variants related to vitamin D metabolism genes have been highlighted, namely rs10741657 (*CYP2R1*), rs10877012 (*CYP27B1*), rs4809959 (*CYP24A1*), and rs731236 (*VDR*); however, since the analyzed variants are located in different cytogenetic locations (*CYP2R1* (11p15.2), *CYP27B1* (12q14.1), *CYP24A1* (20q13.2), and *VDR* (12q13.11) [3], MDR represents an appropriate strategy to evaluate possible epistatic interactions among these unlinked loci. MDR is well-suited for detecting non-additive multilocus effects by classifying multigenotype combinations into binary risk categories, without relying on assumptions about genetic models or chromosomal proximity. This makes it an ideal tool for identifying epistatic interactions and non-additive effects in polygenic traits [16].

Its application is especially valuable when analyzing SNV–SNV interactions across dispersed genomic regions that functionally converge within common metabolic or signaling pathways, such as those involved in vitamin D metabolism and immune regulation. In our study, MDR enabled reduction of the multilocus matrix involving the SNVs in *CYP2R1*, *CYP27B1*, *CYP24A1*, and *VDR* to two informative variants: rs10877012 in *CYP27B1* and rs731236 in *VDR*. These SNVs demonstrated the best genotype-based classification of individuals with either vitamin D deficiency, increased potential genetic susceptibility to developing RA, or higher disease activity, supported by high cross-validation (CVC = 10/10) and balanced testing accuracy (TA = 0.57 for hypovitaminosis D for rs 10877012; TA = 0.58 for RA susceptibility and TA = 0.62 for high clinical activity for rs 731236). Our findings highlight the relevance of the rs10877012 (*CYP27B1*) and rs731236 (*VDR*) variants, whose described molecular effects correspond to the observed clinical phenotypes. Notably, carriers of the GG genotype of rs10877012 (*CYP27B1*) have an increased susceptibility to hypovitaminosis D, reinforcing the gene’s role in regulating serum calcidiol levels. Specifically, the rs10877012 (G>T) variant is located in the 5’-UTR region of *CYP27B1*, approximately 1.2 kb upstream of the transcription start site, and functional analyses using luciferase reporter assays have shown that the G allele (–1260C) reduces reporter activity compared with the T allele, indicating a cis-regulatory effect likely mediated by changes in mRNA stability rather than a direct alteration of promoter binding sites [10]. Given that *CYP27B1* encodes 1α-hydroxylase, the enzyme responsible for activating vitamin D by converting calcidiol to calcitriol, reduced expression would impair local calcitriol production in antigen-presenting cells and T cells. Such a deficiency may impair vitamin D-regulated gene expression networks, including the upregulation of *IL10*, *CTLA4*, and *FOXP3*, while allowing unchecked expression of pro-inflammatory cytokines like IL6 and TNF-α [6,7]. Conversely, the rs731236 *TaqI* variant in the *VDR*, although it represents a synonymous change (C>T), located in exon 9 near the 3’ UTR, can still influence gene function. Synonymous SNVs, despite not altering the amino acid sequence, may affect mRNA splicing, stability, or translational efficiency [3]. Notably, rs731236 has been reported to present a high LD with a functional variant known as “singlet A”, located within the polyadenylation signal region of *VDR* [15]. This haplotype influences the use of alternative poly-A sites, resulting in *VDR* mRNA isoforms with shorter or longer 3’ UTRs. The longer isoform, associated with the C allele, harbors more than 17 adenosine residues and is more stable [15]. Enhanced mRNA stability may lead to increased expression of the soluble VDR isoform, which has been implicated in sustained inflammatory signaling and disrupted immune tolerance mechanisms [14,15,26]. This supports the hypothesis that rs731236 *TaqI VDR* acts as a proxy marker within a functional haplotype, which modulates *VDR* expression post-transcriptionally. Although the rs731236 *TaqI VDR* has not been directly associated with RA susceptibility or disease activity, its genotypes have shown associations with vitamin D levels and bone mineral density in RA patients [27]. Specifically, the CC genotype is associated with an increased risk of osteoporosis, while the TT genotype correlates with higher serum calcidiol levels [28,29]. These findings highlighted the pleiotropic effects of *VDR* variants across musculoskeletal and immune outcomes. These results suggest that regulatory variation in *CYP27B1* and *VDR* can modulate disease-relevant traits through changes in gene expression levels, mRNA processing, or transcript stability. From a broader perspective, these SNVs exemplify how noncoding genetic variation contributes to immune dysregulation by altering key metabolic signaling hubs, such as the vitamin D metabolic pathway. Despite these findings, our study has some limitations. First, the present study does not suggest causality between the variables evaluated; due to its cross-sectional design, it only provides information at a specific time point. Second, the study sample included only Mexican mestizo females from a single geographic region, which may limit the generalizability of the findings to other populations, sexes, or regions with different genetic or environmental backgrounds. Third, although we recorded the use of vitamin D supplements among RA patients, we did not distinguish between specific metabolites administered (calcidiol, calcitriol, or cholecalciferol), which may have influenced serum calcidiol levels and represent a potential confounding factor in the analysis of genetic associations. Additionally, although we collected data on the use of RA treatments (glucocorticoids, methotrexate, and hydroxychloroquine), these variables were recorded categorically (yes/no) and showed an uneven distribution across genotypic and clinical groups. This limitation should be considered when interpreting the observed associations. However, according to our results, the strength of the present study was that the sample of RA patients and CS evaluated was homogeneous in the following characteristics: all SNVs assessed were in HWE; moreover, regarding the study design all participants were female, from the same geographic area, classified as the Mexican mestizo population with three ancestors in the same geographic region, which reduces the bias regarding environmental and genetic factors of ancestry that could influence the results.

Furthermore, while differences in *CYP27B1* and *VDR* expression have been documented in autoimmune and chronic inflammatory diseases such as multiple sclerosis and cancer [30,31], direct transcriptomic evidence in RA remains scarce. Therefore, from the perspective of this study, it will be necessary to assess further in vitro studies the biological effect of the SNVs on vitamin D metabolism pathways and immune regulation, to generate a functional validation that would help to clarify the mechanistic role and impact of these SNVs in the immunometabolic function of vitamin D.

## 5. Conclusions

The GG genotype of rs10877012 *CYP27B1* was significantly associated with susceptibility of having hypovitaminosis D, whereas the CT genotypes of rs731236 *TaqI VDR* confer susceptibility to RA and high clinical disease activity in the Mexican mestizo population. These findings highlight the importance of genetic variation in vitamin D metabolism and signaling pathways as contributors to both vitamin D deficiency and RA pathogenesis, suggesting potential targets for personalized therapeutic interventions and risk stratification in this admixed population.

## Figures and Tables

**Table 1:** Anthropometric, biochemical, clinical, and vitamin D variables from RA patients and CS.

Variable	RA Patients (*n* = 204)	CS (*n* = 204)	*p* Value ^*^
*Anthropometric*			
Age (years) ^a^	48 (28–65)	34 (19–59)	**<0.001**
BMI (kg/m^2^) ^a^	27.2 (19.8–37.9)	24.0 (18.8–34.9)	**<0.001**
*Biochemical*			
Glucose (mg/dL) ^a^	87 (69.9–127)	88.2 (74.4–114.2)	0.9
Cholesterol (mg/dL) ^a^	168.2 (119.9–232)	174.3 (125.3–243.8)	0.2
Triglycerides (mg/dL) ^a^	98.5 (47.0–195)	76.0 (39.4–198.7)	**<0.001**
HDL-C (mg/dL) ^a^	48.9 (29.9–74.9)	51.4 (34.7–75.9)	**0.02**
LDL-C (mg/dL) ^a^	92.1 (40.8–142)	95.2 (61.6–157.2)	**<0.01**
Uric acid (mg/dL) ^a^	3.8 (2.1–6.8)	4.4 (3.1–7.3)	**<0.001**
Albumin (g/dL) ^a^	3.8 (3.3–4.5)	3.8 (3.4–4.42)	0.5
*Clinical*			
Disease duration (years) ^a^	7 (1–25)	-	
Tender joints ^a^	1.5 (0–10)	-	
Swollen joints ^a^	1 (0–8)	-	
DAS28 (ESR) ^a^	3.5 (1.8–6.1)		
Remission (DAS28-ESR <2.6) ^b^	21 (29/136)	-	
Activity (DAS28-ESR >2.6 )^b^	79 (107/136)	-	
CRP (mg/L) ^a^	4.9 (0.5–35.2)	1.2 (0–12.6)	**<0.001**
ESR (mm/h) ^a^	32.5 (8–79)	-	
ACPAs (UI/mL) ^a^	256 (1.5–1173)	-	
RF (UI/mL) ^a^	121.5 (8–607)	-	
*Vitamin D metabolites*			
Calcidiol (ng/mL) ^a^	23.7 (9.4–53.9)	22.9 (10.9–39.4)	0.3
Without hypovitaminosis D ^b^	37 (48/119)	36 (71/119)	0.87
With hypovitaminosis D ^b^	63 (81/206)	64 (125/206)	
*Treatment*			
NSAIDs ^b^	68 (135/198)	-	
Glucocorticoids ^b^	18 (35/193)	-	
Azathioprine ^b^	4 (7/194)	-	
Methotrexate ^b^	85 (170/199)	-	
Chloroquine ^b^	29 (58/199)	-	
Hydroxychloroquine ^b^	8 (15/177)	-	
Sulfasalazine ^b^	71 (142/198)	-	
Vitamin D supplements (Cholecalciferol)	23 (45/197)	-	

^a^ Data provided in median (percentile: p5th–p95th), * *p* value: U Mann–Whitney test. ^b^ Data provided as % (n/N), where n = number of individuals and N = total number evaluated, * *p* value: F-fisher. Bold numbers in column of *p* Values indicate significant differences. BMI: body mass index; HDL-C: high density lipoprotein; LDL: low density lipoprotein; DAS: disease activity score; ESR: erythrocyte sedimentation rate; CRP: C-reactive protein; ACPAs: anti-citrullinated peptide antibodies; RF: rheumatoid factor; and NSAID: non-steroidal anti-inflammatory drugs.

**Table 2:** Interaction of vitamin D metabolism SNVs in determining hypovitaminosis D, RA, and its clinical disease activity.

Susceptibility to Hypovitaminosis D						
Total (n = 324)	Without Hypovitaminosis D (n = 119)	With Hypovitaminosis D (n = 205)	TA	CVC	OR (95% CI)	*p*-Value
*Model 1* *VDR* (rs731236), *CYP2R1* (rs10741657), and *CYP24A1* (rs4809959)	TT or CT in *VDR* (rs731236), plus any genotype in *CYP2R1* (rs10741657), and AA or GG in *CYP24A1* (rs4809959)	Any other combination	0.51	4/10	2.9 (1.4–6.4)	**<0.01**
*Model 2* *CYP27B1* (rs10877012) and *CYP24A1* (rs4809959)	GT in *CYP27B1* (rs10877012), and AA in *CYP24A1* (rs4809959)	Any other combination	0.50	5/10	2.2 (1.1–4.1)	**0.02**
*Model 3* *CYP27B1 (rs10877012)*	GT in *CYP27B1* (rs10877012)	GG or TT in *CYP27B1*	0.57	10/10	1.7 (1.1–2.7)	**0.02**
**Susceptibility to rheumatoid arthritis**						
**Total (n = 402)**	**Low** **(n = 199)**	**High** **(n = 204)**	**TA**	**CVC**	**OR (95% CI)**	***p*** **-Value**
*Model 1* *VDR* (rs731236), *CYP2R1* (rs10741657), and *CYP27B1* (rs10877012)	TT in *VDR* (rs731236), plus GG in *CYP2R1* (rs10741657)*, and* plus GG or GT in *CYP27B1* (rs10877012)	Any other combination	0.57	7/10	2.9 (1.7–4.9)	**<0.001**
*Model 2* *VDR* (rs731236), and *CYP27B1* (rs10877012)	TT in *VDR* (rs731236) plus GG and GT in *CYP27B1* (rs10877012)	Any other combination	0.61	10/10	2.5 (1.6–3.8)	**<0.001**
*Model 3* *VDR* (rs731236)	TT in *VDR* (rs731236)	CT or CC in *VDR*	0.58	10/10	1.9 (1.3–2.8)	**<0.01**
**Susceptibility to high clinical disease activity (DAS28-ESR)**						
**Total (n = 134)**	**Disease remission** **(n = 105)**	**Disease activity** **(n = 29)**	**TA**	**CVC**	**OR (95% CI)**	***p*** **-Value**
*Model 1* *VDR* (rs731236), *CYP2R1* (rs10741657), and *CYP27B1* (rs10877012)	TT in *VDR* (rs731236), plus GG in *CYP2R1* (rs10741657), and GG in *CYP27B1* (rs10877012)	Any other combination	0.57	10/10	10.7 (1.9–58.7)	**<0.01**
*Model 2* *VDR* (rs731236) and *CYP2R1* (rs10741657)	TT in *VDR* (rs731236) and GG in *CYP2R1* (rs10741657)	Any other combination	0.50	7/10	2.9 (1.1–8.12)	**0.03**
*Model 3* *VDR* (rs731236)	TT in *VDR* (rs731236)	CT or CC in *VDR*	0.62	10/10	2.8 (1.2–6.6)	**0.01**

TA: testing accuracy; CVC: cross-validation consistency; OR: odds ratio; CI: confidence interval. Bold numbers in column of *p* Value indicate significant differences.

**Table 3:** Association of (rs10741657) *CYP2R1*, (rs10877012) *CYP27B1,* (rs4809959) *CYP24A1,* and (rs731236) *VDR* genotypes with hypovitaminosis D, rheumatoid arthritis, and its clinical disease activity susceptibility.

	Hypovitaminosis D (Both Groups)					RA Disease Susceptibility					Clinical Disease Activity (DAS28-ESR)				
SNV	With Hypovitaminosis D (n = 119)	Without Hypovitaminosis D (n = 205)	*p* Value	OR (95% CI)	*p* Value	RA (n = 199)	CS (n = 204)	*p* Value	OR (95% CI)	*p* Value	Activity (n = 105)	Remission (n = 29)	*p* Value	OR (95% CI)	*p* Value
rs10741657 *CYP2R1*			0.8					0.8					0.8		
AA ^§^	10 (12)	11 (23)		1		12 (23)	10 (21)		1		11 (12)	14 (4)		1	
AG	50 (59)	46 (94)		1.2 (0.5–2.8)	0.6	47 (94)	47 (95)		0.9 (0.4–1.8)	0.8	45 (47)	48 (14)		1.1 (0.2–4.5)	0.9
GG	40 (48)	43 (88)		1.0 (0.4–2.5)	0.9	41 (82)	43 (88)		0.8 (0.4–1.7)	0.6	44 (46)	38 (11)		1.4 (0.3–5.8)	0.6
rs10877012 *CYP27B1*			**0.04**					0.08					0.8		
GG	55 (66)	41 (84)		1.8 (1.1–3.0)	**0.01**	47 (94)	44 (89)		1.3 (0.8–2.0)	0.2	47 (49)	45 (13)		0.9 (0.3–2.6)	0.9
GT ^§^	37 (44)	51 (104)		1		41 (81)	49 (101)		1		41 (43)	38 (11)		1	
TT	8 (9)	8 (17)		1.2 (0.4–3.2)	0.6	12 (24)	7 (14)		2.1 (0.98–4.8)	0.04	12 (13)	17 (5)		0.7 (0.2–2.9)	0.5
rs4809959 *CYP24A1*			0.5					0.2					0.7		
AA ^§^	35 (42)	41 (85)		1		43 (85)	36 (74)		1		43 (45)	45 (13)		1	
AG	46 (55)	41 (85)		1.3 (0.8–2.2)	0.3	34 (67)	42 (85)		0.7 (0.4–1.1)	0.1	33 (35)	38 (11)		0.9 (0.3–2.6)	0.8
GG	18 (22)	18 (35)		1.2 (0.6–2.5)	0.5	23 (47)	22 (45)		0.9 (0.5–1.5)	0.7	24 (25)	17 (5)		1.4 (0.4–5.7)	0.5
rs731236 *VDR*			0.6					**<0.01**					**0.02**		
CC	9 (11)	6 (13)		1.4 (0.5–3.7)	0.4	8 (16)	5 (11)		2.1 (0.9–5.4)	0.06	10 (10)	14 (4)		1.2 (0.3–6.1)	0.7
CT	43 (51)	46 (94)		0.9 (0.6–1.5)	0.7	54 (107)	41 (83)		1.9 (1.2–2.9)	**<0.01**	57 (60)	27 (8)		3.6 (1.3–10.7)	**<0.01**
TT ^§^	48 (57)	48 (98)		1		38 (75)	54 (110)		1		33 (35)	59 (17)		1	

RA: rheumatoid arthritis; CS: control subject; OR: odds ratio; CI: confidence interval; ^§^: reference genotype; Bold numbers in column of *p* Value indicate significant differences.

## Data Availability

The data used to support the findings of this study are available from the corresponding author upon reasonable request.

## Abbreviations

The following abbreviations are used in this manuscript:

ACR	American College of Rheumatology
ACPAs	Anti-citrullinated peptide antibodies
BMI	Body mass index
CI	Confidence interval
CRP	C-reactive protein
CVC	Cross-validation consistency
CYP2R1	Cytochrome P450 family 2 subfamily R member 1
CYP24A1	Cytochrome P450 family 24 subfamily A member 1
CYP27B1	Cytochrome P450 family 27 subfamily B member 1
DAS28	Disease activity score in 28 joints
ESR	Erythrocyte sedimentation rate
HDL-C	High-density lipoprotein cholesterol
CS	Control subjects
LDL-C	Low-density lipoprotein cholesterol
MDR	Multifactor dimensionality reduction
OR	Odds ratio
PCR	Polymerase chain reaction
RA	Rheumatoid arthritis
RF	Rheumatoid factor
SNV	Single nucleotide variant
TA	Testing accuracy
UTR	Untranslated region
VDR	Vitamin D receptor
VDRE	Vitamin D response element
